# Clinical Performance of Posterior Indirect Resin Composite Restorations with the Proximal Box Elevation Technique: A** Prospective Clinical Trial up to 3 Years**

**DOI:** 10.3290/j.jad.b4908449

**Published:** 2024-01-26

**Authors:** Burcu Gözetici-Çil, Funda Öztürk-Bozkurt, Gencay Genç-Çalışkan, Burcu Yılmaz, Nurcan Aksaka, Mutlu Özcan

**Affiliations:** a Assistant Professor, Department of Restorative Dentistry, Istanbul Medipol University, School of Dentistry, Istanbul, Turkey. Conceptualization, methodology, operator, data curation, wrote the manuscript.; b Professor, Department of Restorative Dentistry, Istanbul Medipol University, School of Dentistry, Istanbul, Turkey. Funding acquisition, supervision, reviewed the manuscript.; c Assistant Professor, Department of Restorative Dentistry, Istanbul Medipol University, School of Dentistry, Istanbul, Turkey. Data curation, observer, reviewed the manuscript.; d Assistant Professor, Department of Prosthodontics, Istanbul Medipol University, School of Dentistry, Istanbul, Turkey. Data curation, observer, reviewed the manuscript.; e Assistant Professor, Department of Periodontology, Istanbul Medipol University, School of Dentistry, Istanbul, Turkey. Data curation, observer, reviewed the manuscript.; f Professor, Clinic for Reconstructive Dentistry, Division of Dental Biomaterials, Center for Dental Medicine, University of Zurich, Zurich, Switzerland. Methodology, funding acquisition, supervision, reviewed and edited the manuscript.

**Keywords:** adhesive dentistry, clinical trial, dental materials, indirect resin composite, survival, proximal box elevation

## Abstract

**Purpose:**

The study evaluated the clinical performance of partial indirect resin composite (PIRC) restorations with “proximal box elevation” (PBE) placed in molars.

**Materials and Methods:**

Sixty-three patients received 80 posterior PIRC (SR Nexco, Ivoclar Vivadent) restorations. Large posterior defects with cuspal loss and deep cervical margins were included in the study. PBE was performed prior to preparation and impression making. Two independent observers evaluated the restorations using the FDI criteria (scores 1-5) for esthetic, functional, and biological properties. Patients were recalled at 6 months and 1, 2, and 3 years. Overall success rates were calculated (Kaplan-Meier analysis) and compared (log-rank tests) according to baseline variables. The impact of the baseline variables on the failure of the restorations was analyzed (multiple proportional Cox regression).

**Results:**

Seventy-nine (98.7%), 69 (88.4%), 66 (92.9%), 44 (86.2%) and 45 (91.8%) PIRCs completed their follow up at baseline, 6 months, and 1, 2 and 3 years, respectively. In total, 10 failures were observed: 5 with partial loss, 4 with material chipping, and one with secondary caries, yielding an overall success rate of 87.5% and a survival rate of 93.8%, with a mean observation time of 26.5 ± 13.6 months.

**Conclusions:**

PIRCs with PBE demonstrated a high survival rate and satisfactory esthetic properties. Failure was less likely for PIRC restorations with partial cuspal coverage (onlay) compared to full cuspal coverage (overlay).

Indirect restorations are recommended for large posterior defects in order to prevent problems related to polymerization shrinkage and promote reinforcement of teeth compromised with caries or fractures.^22,32^ Onlays (covering at least one cusp), and overlays (covering all cusps) made of composite, ceramic, or hybrid ceramics are proposed to be more conservative options than conventional complete crowns.^27,32^ The survival rate of resin-composite onlays was reported to be 100% and 91.1% and after 3 and 5 years, respectively.^5,10^ Similarly, medium-term studies (2–5 years) showed a survival rate of 91%–100% for ceramic onlays.^1^

The mechanical properties of ceramics make them better able to resist compressive forces and transfer less stress to tooth structure than resin composites.^34^ However, they can induce enamel wear on antagonist teeth.^4,24^ Hybrid ceramics and resin-based materials might be more advantageous than ceramics, not only due to an easier manufacturing process but because their physical and mechanical properties are closer to natural tooth structure. In previous studies, computer-aided designed/computer-aided manufacturing (CAD/CAM) hybrid ceramic and resin-based composite onlays were shown to perform similar to ceramics, but with better esthetic properties and fracture resistance.^11,12^

Apart from the material choice, difficulties in tooth preparation, impression taking, adhesive cementation of the indirect restoration, and finishing and polishing in subgingival areas can be eliminated by using the proximal box elevation (PBE) technique. PBE refers to relocation of the subgingival cervical margin supragingivally, using a resin composite material in the deepest parts of the proximal areas of the preparation.^6^ This technique was first introduced as “cervical margin relocation” in 1998 and later on widely termed “deep margin elevation”.^9,25^ The term PBE was proposed in 2017 to define proximal margin elevation for Class-II cavities.^6^ Recently, a long-term retrospective study reported high survival rates for both ceramic (96.5%) and resin composite restorations (94.3%) using deep margin elevation.^4^

The results of the studies cited above seem to be promising for a shift of choice from ceramic to resin-based materials and application of PBE. However, there is lack of prospective clinical trials with a defined protocol to provide evidence for the clinical performance of partial indirect resin composite (PIRC) restorations in conjunction with PBE. Therefore, the aim of this prospective clinical trial was to evaluate the clinical performance of laboratory-processed PIRC restorations with the PBE technique. The primary outcome of the study was to evaluate survival and quality of the restorations based on FDI criteria^18^ at baseline, 6 months, and 1, 2, and 3 years. The secondary outcome was to investigate the impact of baseline variables on the success of the PIRC restorations. The first null hypothesis was that there would be no differences in quality of surviving indirect restorations in terms of esthetic, functional, and biological properties over time. The second null hypothesis tested was that there would be no differences in the success of PIRC restorations with one-sided or two-sided PBE, partial (onlay) or full cuspal coverage (overlay), endodontically treated or vital teeth, need for surgical exposure of subgingival cervical margins or not.

## Materials and Methods

This prospective single-arm clinical trial was registered (clinicaltrials.gov, registration No. NCT 03832829) and approved by the University Ethics Committee (protocol No. 10840098-604.01.01-E.34133, approval date:15.08.2018). The participants were selected from patients referred to the Restorative Dentistry Department of the University Clinic due to restoration need. All participants provided written informed consent. Teeth with extensive substance loss (N = 472) were evaluated for eligibility to be included in the study between September 2018 and April 2022. Of these teeth, 392 were excluded as they did not meet the inclusion criteria.

### Exclusion and Inclusion Criteria

- Inclusion criteria: molars with large defects including deep cervical margin/s on the mesial and/or distal side and at least loss of one cusp; teeth with antagonist natural dentition and at least one proximal contact with a natural adjacent tooth; vital or endodontically treated; patients over 18 years of age in good general health.
- Exclusion criteria: Untreated periodontal disease with horizontal and/or vertical mobility, poor endodontic prognosis or pulp exposure that might require further root canal therapy; presence of severe wear facets; pregnancy or breast feeding; patients who declined to participate.

The sample size calculation was based on the difference between success rates of indirect resin composite restorations without PBE of 82.1%^13^ and with PBE of 96%^8^ within the same observation period of 5 years, at α = 5% with a power of 80%. This indicated a need for 43 PIRC restorations. Taking into consideration the possibility of drop-outs or no-shows, 80 PIRC restorations were included.

Clinical treatments were carried out by an operator with over 15 years of experience in restorative dentistry since graduation. The operator was trained in all clinical procedures prior to the study. Pre-operative radiographs and occlusal photographs were made of each case. Initial periodontal therapy including scaling and root planing was performed 2 weeks prior to the cavity preparation, if required. No periodontal surgery was performed to expose deep cervical margins. Operative procedures were performed under local anesthesia.

Age, gender, type of tooth, and reason for restoration was recorded prior to the operative procedure. After removal of defective old restorations and carious tissue, remaining cavity walls and compromised cusps with less than 2–3 mm thickness were reduced occlusally by 1.5 to 2 mm. The PBE elevation sides, cusps, and surfaces to be restored were recorded. Thereafter, the periodontal status of the tooth was evaluated and classified as grade 1 (no need for periodontal surgery), grade 2 (need for soft tissue removal), or grade 3 (need for crown lengthening) considering the biological width and isolation conditions.^33^

The circumferential matrix band (Adapt Super Cap Matrix, Kerr-Hawe; Bioggio, Switzerland) was placed and fixed with wooden wedges prior to PBE and resin composite build-up procedure. The operative field was cleaned with an air/water spray, gently air dried, and then carefully isolated with cotton rolls and suction. No rubber-dam or retraction cord was used, since proper isolation could not be provided at deep proximal margins. A three-step adhesive (Syntac Primer/Adhesive System, Heliobond, Ivoclar Vivadent; Schaan, Liechtenstein), flowable composite (Tetric-N Flow, Ivoclar Vivadent) with a maximum thickness of 1–1.5 mm, and resin composite (Tetric-N Ceram, Ivoclar Vivadent) were used in sequence according to the manufacturer’s instructions. After the composite build-up procedure was completed, the matrix band was removed, and the final restoration was further photo-polymerized (LED.B, Guilin Woodpecker Medical Instrument; Guilin, Guangxi, China) for 40 s from the buccal, palatinal/lingual, and occlusal aspects. Bevel preparation was performed coronal to the equatorial tooth line and butt-joint preparation (1–1.2 mm, maximum 1.5 mm) apical to the equatorial line using a shoulder bur. Fine-grit diamond burs and coarse to fine abrasive disks (Sof-Lex, 3M Oral Care; St Paul, MN, USA) were used in sequence for finishing and polishing the PBE sides. Thus, the cavity consisted of a box configuration in the gingival margins and inclined plane in cuspal-coverage areas. Interior walls diverging 6 to 10 degrees with rounded interior angles were obtained using a conical medium-grit diamond bur. Impressions were made with the double impression technique using putty (Zetaplus, Zhermack; Badia Polesine, Italy) and low-viscosity condensation silicone material (Oranwash L, Zhermack), after which provisional resin restorations were placed (Clip, VOCO; Cuxhaven, Germany).

One experienced dental technician fabricated all of the indirect restorations using a laboratory processed resin composite material (SR Nexco, Ivoclar Vivadent) and a laboratory polymerization device (GC Labolight LV-III, GC; Tokyo, Japan) according to the respective manufacturer’s instructions. All restorations were permanently bonded within 1 week. Prior to cementation, transparent molar bands (Hawe Molar Matrices Transparent, Kerr-Hawe) were placed and fixed with wooden wedges to provide better adaptation on proximal aspects and prevent formation of negative/positive steps at the margins. After the restoration fit was checked, the cavity was etched for 30 s on enamel margins and 15 s on internal surfaces with 37% phosphoric acid (Total Etch, Ivoclar Vivadent), rinsed thoroughly with water, and air dried. Isolation was provided by cotton rolls and suction. The primer and adhesive (Syntac Primer/Adhesive/Heliobond System, Ivoclar Vivadent) were used in sequence for conditioning the internal surfaces of the cavity without photo-polymerization to avoid inadequate fitting of the restoration. A dual-polymerizing resin cement (Variolink-N, Ivoclar Vivadent) was used for cementation. The internal surfaces of the restorations were coated with silane coupling agent (Monobond N, Ivoclar Vivadent). Restorations were placed under slight pressure and photopolymerized with an LED device (LED.B, Guilin Woodpecker Medical Instrument) emitting 1200 W/cm^2^ for 40 s from three sides covered with glycerin gel (Liquid strip, Ivoclar Vivadent). Necessary adjustments were made using fine-grit diamond burs, disks (Sof-Lex 3M Oral Care), and strips.

Two calibrated evaluators (with over 10 years of experience in prosthodontics and restorative dentistry since graduation) independently evaluated the PIRC restorations at baseline (2 weeks after treatment), 6 months, and 1, 2, and 3 years according to FDI World Dental Federation criteria (Table 1). For the criteria regarding marginal quality, the semi-quantitative clinical evaluation (SQUACE) method was carried out to assess the proportion of the total length of the restoration margin scored as not being clinically excellent (2-5).^19^ Disagreements in evaluations were discussed between the evaluators and a consensus was reached. Digital photographs were taken with a standard magnification. The radiographic examinations were performed immediately after the treatment to check for overhangs, gaps, and excessive cement in the cervico-approximal region.

**Table 1:** FDI evaluation criteria and ratings

	Criteria	Description of the criteria	Rating
**Esthetic Properties**	FDI 1. Surface Luster	Luster comparable to enamel.	1
		Slightly dull not noticeable from speaking distance (1); some isolated pores (2).	2
		Dull surface but acceptable if covered with film of saliva (1); multiple pores on more than one third of the surface (2).	3
		Rough surface not masked by saliva, simple polishing is not sufficient, further intervention is necessary (1); voids (2).	4
		Very rough, unacceptable plaque retentive surfaces.	5
	FDI 2. Staining (FDI 2a. Surface staining) (FDI 2b. Marginal staining)	No staining.	1
		Minor staining, easily removable by polishing.	2
		Moderate staining that may also present on other teeth, not esthetically unacceptable.	3
		Unacceptable staining and major intervention necessary for improvement.	4
		Severe staining, not accessible for intervention.	5
	FDI 3. Color match and translucency	Good color match, no difference in shade and/or translucency.	1
		Minor deviations in shade and/or translucency.	2
		Distinct deviation but acceptable. Does not affect esthetics. More opaque (1) translucent (2), brighter (3) or darker (4).	3
		Localized clinical deviation that can be corrected by repair. Too opaque (1), translucent (2), bright (3) or dark (4).	4
		Unacceptable, replacement necessary.	5
**Functional Properties**	FDI 5. Fracture of material and retention	No fracture/cracks.	1
		Small hairline crack.	2
		Two or more larger hairline cracks and/or material chip not affecting marginal integrity or approximal contact.	3
		Material chip fractures which damage marginal quality or approximal contacts (1); bulk fractures with partial loss less than half of the restoration (2).	4
		Partial/complete loss of restorations or multiple fractures.	5
	FDI 6. Marginal adaptation	Harmonious outline, no gaps, no white or discolored lines.	1
		Marginal gap (<150 µm), white lines (1); small marginal fracture removable by polishing (2); slight ditching, slight step/flashes, minor irregularities (3).	2
		Gap < 250 µm not removable (1); several small marginal fractures (2); major irregularities, ditching or flash, steps (3).	3
		Gap > 250 µm or dentin/base exposed (1); severe ditching or marginal fractures (2); larger irregularities or steps, repair necessary (3).	4
		Restoration (complete or partial) is loose but in situ (1); generalized major gaps or irregularities (2).	5
	FDI 7. Occlusal contour and wear (qualitatively)	Physiologic wear equivalent to enamel.	1
		Normal wear only slightly different from that of enamel.	2
		Different wear rate to enamel but within the biological variation.	3
		Wear considerably exceeds normal enamel wear; or occlusal contact points are lost.	4
		Wear is excessive.	5
	FDI 8. Approximal anatomical form (FDI 8a. Contact point) (FDI 8b. Contour)	Normal contact point (floss or 25 µm metal blade can pass) and contour.	1
		Contact slightly too strong but no disadvantage (floss or 25 µm metal blade can only pass with pressure); slightly deficient contour.	2
		Somewhat weak contact, no indication of damage to tooth, gingiva or periodontal structures; 50 µm metal blade can pass; visibly deficient contour.	3
		Too weak and possible damage due to food impaction; 100 µm metal blade can pass; inadequate contour. Repair possible.	4
		Too weak and/or clear damage due to food impaction and/or pain/gingivitis, insufficient contour requires replacement.	5
	FDI 10. Patient’s view	Entirely satisfied with esthetics and function.	1
		Satisfied esthetics (1); function, eg, minor roughness (2).	2
		Minor criticism but no adverse clinical effects; esthetic shortcomings (1); some lack of chewing comfort (2); unpleasant treatment procedure (3).	3
		Desire for improvement esthetics (1); function, eg, tongue irritation (2). Reshaping of anatomic form or refurbishing is possible.	4
		Completely dissatisfied and/or adverse effects, including pain.	5
**Biological Properties**	FDI 11. Postoperative (hyper-) sensitivity and tooth vitality	No hypersensitivity; normal vitality.	1
		Minor hypersensitivity for a limited period of time; normal vitality.	2
		Moderate hypersensitivity (1); delayed/mild sensitivity; no subjective complaints, no treatment needed (2).	3
		Intense hypersensitivity (1); delayed with minor subjective symptoms (2); no clinical detectable sensitivity (3). Intervention necessary but not replacement.	4
		Intense, acute pulpitis or nonvital tooth. Endodontic treatment necessary and restoration has to be replaced.	5
	FDI 12. Recurrence of caries, erosion, abfraction	No secondary or primary caries.	1
		Small and localized. Demineralization (1), erosion (2) or abfraction (3).	2
		Larger areas of demineralization (1), erosion (2) or abrasion/abfraction (3), dentin not exposed. Only preventive measures necessary.	3
		Caries with cavitation and suspected undermining caries (1); erosion (2), abrasion/abfraction (3) in dentin. Localized and accessible can be repaired.	4
		Deep caries or exposed dentin that is not accessible for repair of restoration.	5
	FDI 13. Tooth integrity (enamel cracks, tooth fractures)	Complete integrity.	1
		Small marginal enamel fracture <150 µm (1); hairline crack in enamel <150 µm (2).	2
		Marginal enamel defect (1) or crack <250 µm (2); enamel chipping (3) or multiple cracks (4).	3
		Major marginal enamel defects; gap >250 µm or dentin base exposed (1); large cracks >250 µm probe penetrates (2); large enamel chipping or wall fracture (3).	4
		Cusp or tooth fracture.	5

In case of fracture, the protocol for repair process was followed. The defective part of the restoration and remaining sharp edges were removed using a high-speed rotary instrument under water cooling with diamond burs. No retention grooves or bevels were made. The matrix band (Adapt Super Cap Matrix, Kerr-Hawe) was placed and fixed with wooden wedges. The same adhesive and restorative materials used in PBE procedure were applied. Additionally, a silane coupling agent was applied on internal resin composite surfaces after etching.

The baseline variables were defined as cuspal coverage (onlay/overlay), proximal box elevation (one/two sides), endodontic treatment (yes/no), and periodontal status (Grade 1/2/3). Overall cumulative survival and success of the restorations were calculated using the Kaplan-Meier analysis. The Friedman test was used to evaluate differences in the quality of the restorations over time to test the first null hypothesis. The survival of PIRC restorations was compared according to baseline variables using Log Rank (Mantel-Cox) tests to test the second null hypothesis. A multiple proportional Cox regression model was used to analyze the impact of the baseline variables on the failure of the PIRC restorations. The data were analyzed using SPSS software (Version 23.0, IBM; Armonk, NY, USA). For all tests, α was set at 0.05 and the unit of analysis was the tooth.

## Results

**Fig 1:**
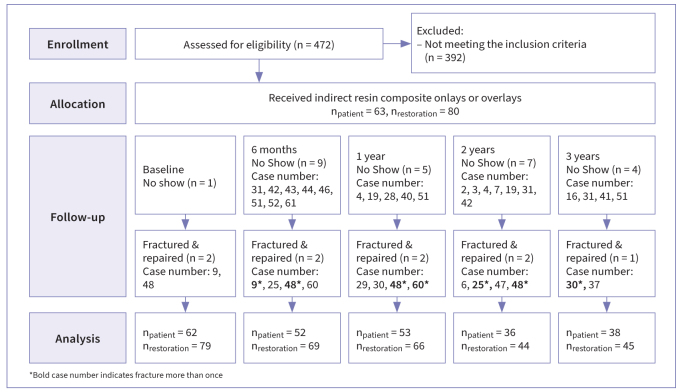
Study flow chart.

A total of 80 PIRC restorations in 63 patients (26 males and 37 females; mean age 27.1 years; range: 18–48 years) were included in the study. Approximately 76% of the patients received one treatment, 21% received two treatments and 3% received three treatments. In total, 69 (88.4%), 66 (92.9%), 44 (86.2%) and 45 (91.8%) PIRC restorations were followed-up, whereas 9, 5, 7 and 4 patients did not attend follow-ups due to various reasons such as the Covid-19 pandemic and relocation at 6 months, 1 year, 2 and 3 years, respectively (Fig 1). Some of the restorations could be evaluated longer than 3 years in terms of survival and success at extra appointments, upon request of the patient for another dental treatment need. Thus, the mean observation time was 26.5 ± 13.6 months. All baseline characteristics are listed in Table 2. Characteristics of PIRC restorations according to substance loss at baseline are shown in Table 3 with related success and failure rates.

**Table 2:** Description of baseline characteristics

Variables		n	%
Gender	Female	37	58.7
	Male	26	41.3
Type of tooth	First maxillary molar	19	23.75
	Second maxillary molar	7	8.75
	First mandibular molar	47	58.75
	Second mandibular molar	7	8.75
Reason for large defect	Need of replacement	78	97.5
	Primary caries	2	2.5
Vitality of the tooth	Vital	20	25
	Endodontically treated	60	75
Cuspal coverage	Onlay	38	47.5
	Overlay	42	52.5
PBE sides	One-sided	42	52.5
	Two-sided	38	47.5
Periodontal status	Grade 1	45	56.3
	Grade 2	14	17.5
	Grade 3	21	26.3

**Table 3:** PIRC restorations according to size of the restoration in terms of cusps and surfaces involved with related success and failure rates

Type of PIRC restoration	Description of size of PIRC restoration	Total n	Success n (%)	Failure n (%)
Partial Coverage n = 38	2 cusps and 3 surfaces (MOP, MOB, DOL, DOP, DOB)	7	7 (100)	0 (0)
	2 cusps 4 surfaces (MODB, MOBL*, MOBP, MODL, DOBP)	24	23 (95.8)	1 (4.2)
	3 cusps 3 surfaces (MOP*)	1	0 (0)	1 (100)
	3 cusps 4 surfaces (MOBP, MODP, MODB, DOBL)	6	6 (100)	0 (0)
Full Coverage n = 42	3 surfaces (MOB*, DOL, DOB*)	5	3 (60)	2 (40)
	4 surfaces (MODL*, MODP*, MODB, MOBL, MOBP, DOBP, DOBL)	37	31 (83.7)	6 (16.2)

M: mesial; D: distal; O: occlusal; B: buccal; P: palatinal; L:lingual. *Bold case indicates the restored surfaces of PIRCs with failure

**Fig 2:**
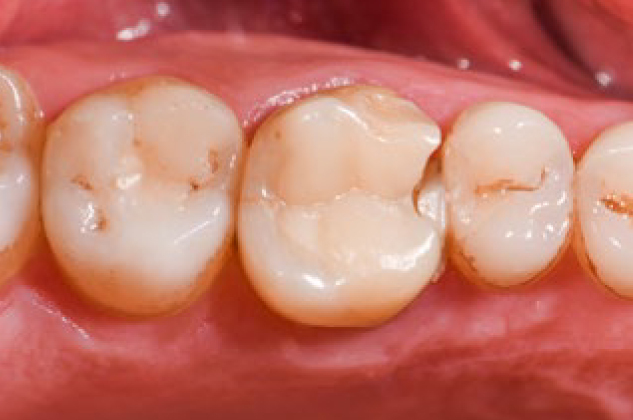
Representative photograph of an onlay restoration on endodontically treated tooth 26 with partial loss (FDI 5.5) after 3 years.

A total of 10 restorations were scored as unacceptable, mainly due to fracture (n = 9), followed by secondary caries (n = 1) within the observation period (range 6–46 months). Five restorations received a score of 5, and 5 restorations received a score of 4, yielding an overall success rate of 87.5% and survival rate of 93.7%. Representative photographs of the fractured PIRC restorations are depicted in Fig 2.

**Fig 3:**
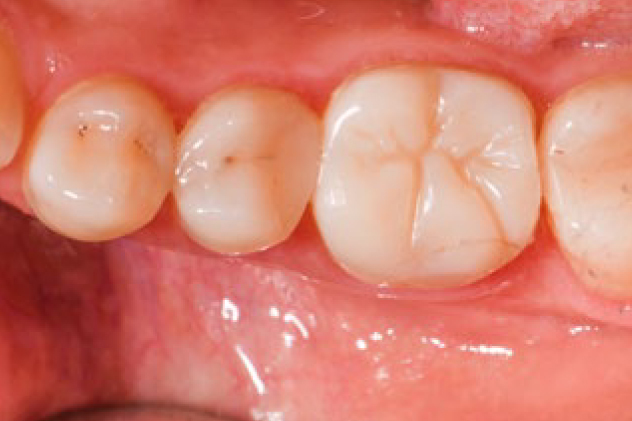
Small hairline crack (FDI 5.2) was detected at 1 year and remained stable at 3 years without further fracture on endodontically treated tooth 46.

**Fig 4:**
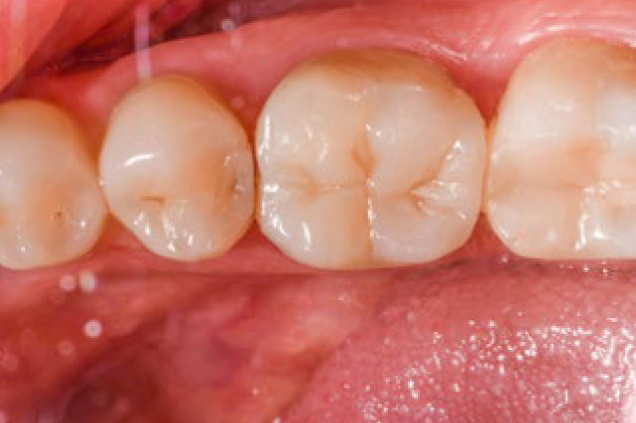
Representative photograph of an overlay restoration on endodontically treated tooth 36 with a somewhat weak contact (FDI 8a.3).

**Fig 5:**
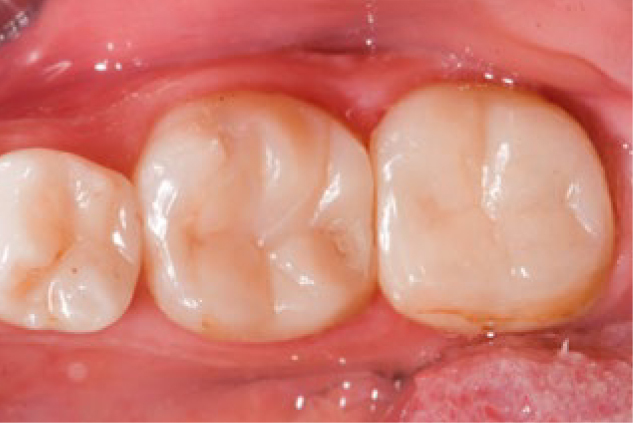
Representative photograph of an onlay on vital tooth 36 and overlay on vital tooth 37, with minor marginal staining (FDI 2b.2) and slight ditching for marginal adaptation (FDI 6.2).

**Fig 6:**
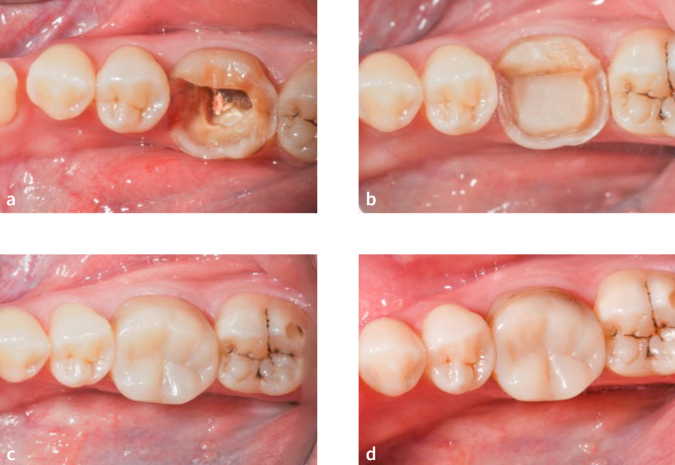
Representative photographs of an overlay on endodontically treated tooth 36 with a) cavity after removal of defective restoration and caries; b) PBE and preparation; c) at baseline; d) secondary caries (FDI 12.4.1) detected at 3 years.

**Fig 7:**
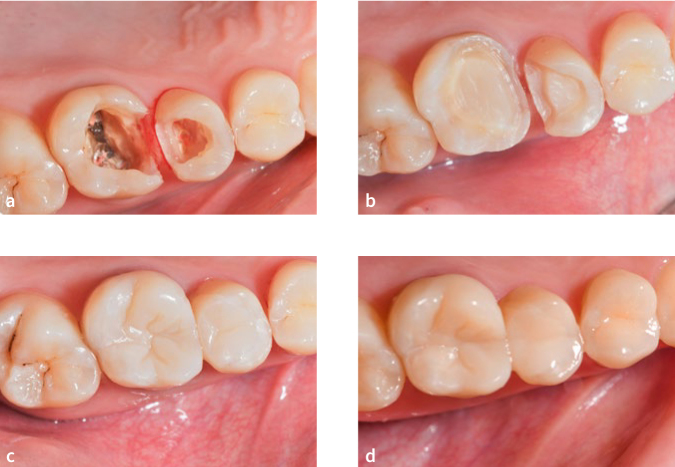
Representative photographs of an onlay restoration on endodontically treated tooth 16 scored as clinically excellent according to FDI criteria. a) cavity after removal defective restoration and caries; b) PBE and preparation; c) at baseline; d) at 3 years.

**Fig 8:**
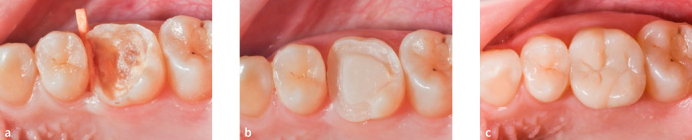
Representative photographs of an onlay restoration on vital tooth 16 scored as clinically excellent according to FDI criteria. a) cavity after removal of defective restoration and caries; b) PBE and preparation; c) at 3 years.

Based on the primary outcome of the study, minor changes were observed in the quality of surviving restorations in terms of esthetic, functional and biological properties. Table 4 shows the FDI criteria for esthetic, functional and biological properties of the restorations. Small hairline cracks were detected in two restorations: one at 6 months and one at 1 year (Fig 3). Three restorations showed chipping that did not affect marginal integrity or approximal contact at 6 months; one at 1 year and one at 2 years. Small marginal fractures detected as irregularities and ditching were observed in one case at baseline and remained unchanged. Acceptable contact-point loss (FDI 8a.3) was observed in six restorations: three at 6 months, one at 2 years and two at 3 years (Fig 4). The proportion of slight ditching or minor irregularities (FDI 6.2) at the margins increased over time (p = 0.002, p < 0.01). Minor marginal staining scores (FDI 2b.2) increased (p < 0.001), whereas minor deviations in surface staining (p = 0.236; p > 0.05), surface luster (p = 0.472; p > 0.05) and color and translucency (p = 0.092; p > 0.05) remained unchanged over time. Representative photographs of PIRC restorations with minor deviations after 3 years are shown in Fig 5. Minor (FDI 11.2) to moderate (FDI 11.3) hypersensitivity was observed in seven cases at baseline and 2 cases at 6 m. One restoration with secondary caries required intervention at 3 years (Fig 6a-d). The mean SQUACE scores for slight discoloration, fracture, gap and secondary caries after 3 years were 9.3% (2–30%), 10% (2–20%), 9.8% (2–40%), and 3%, respectively. Figures 7 (a–d) and 8 (a–c) present representative photographs of PIRC the restorations after 3 years with excellent FDI scores.

**Table 4:** Distribution of the acceptable (1-3) and unacceptable FDI scores (4 and 5) of esthetic, functional and biological properties according to follow-ups

Category	FDI Criteria	Score	Baseline n = 79	6 months n = 69	1 year n = 66	2 years n = 44	3 years n = 45
Esthetic properties	FDI 1	1	74	64	59	40	40
		2	5	5	7	4	5
	FDI 2a	1	79	69	64	40	42
		2	-	-	2	4	3
	FDI 2b	1	79	69	64	37	29
		2	-	-	2	7	16
	FDI 3	1	62	58	62	41	43
		2	15	7	4	2	1
		3	2	4	-	1	1
Functional properties	FDI 5	1	76	63	60	40	42
		2	-	1	2	1	1
		3	1	3	2	1	1
		4	1	1	1	-	1
		5	1	1	1	2	-
	FDI 6	1	73	60	57	37	35
		2	3	6	6	5	9
		3	1	1	1	1	-
		4	1*	1*	1*	-	1*
		5	1*	1*	1*	1*	-
	FDI 8a	1	78	65	64	42	42
		2	-	-	1	-	-
		3	-	3	-	1	3
		5	1*	1*	1*	1*	-
Biological Properties	FDI 11	1	72	67	66	44	45
		2	6	2	-	-	-
		3	1	-	-	-	-
	FDI 12	1	79	69	66	44	44
		4	-	-	-	-	1
	FDI 13	1	79	69	66	44	44
		4	-	-	-	-	1

*Related with fractures.

**Fig 9:**
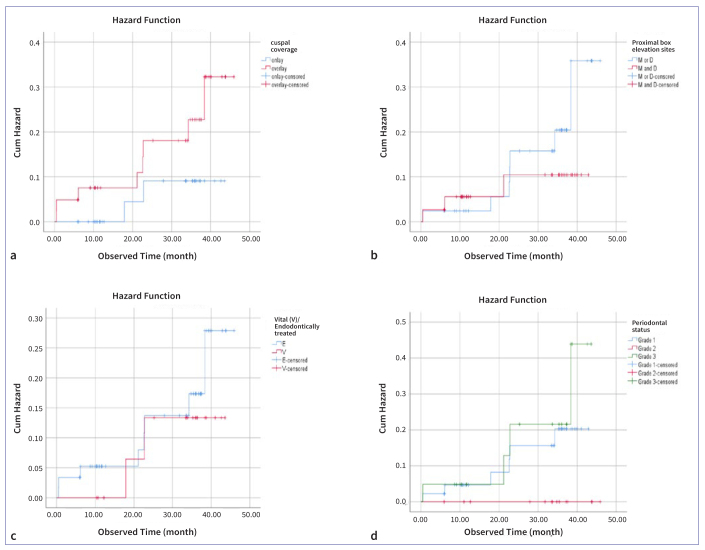
Hazard plots of 80 PIRC restorations using PBE compared with log-rank (Mantel-Cox) tests according to A) cuspal coverage (onlay: n = 38, events n = 2, failure 5.3%; overlay n = 42, events n = 8, failure 19%, p = 0.136; p > 0.05); B) PBE sides (one-sided: n = 42, events n = 7, failure 16.7%; two-sided n = 38, events n = 3, failure 7.9%, p = 0.380; p > 0.05); C) endodontic treatment (vital: n = 20, events n = 2, failure 10%; non-vital n = 60, events n = 8, failure 13.3%, p = 0.555; p > 0.05); and D) periodontal status (grade 1: n = 45, events n = 6, failure 13.3%; grade 2: n = 14, events n = 0, failure 0%; grade 3: n = 21, events n = 4, failure 19%, p = 0.242; p > 0.05).

The Log-Rank (Mantel-Cox) test showed no statistically significant difference in the success of restorations between onlay and overlay restorations (p = 0.136; p > 0.05); one-sided and two-sided PBE (p = 0.380; p > 0.05); vital and endodontically treated teeth (p = 0.555; p > 0.05); grade 1, grade 2 and grade 3 periodontal status (p = 0.242; p > 0.05). Failure curves are given in Fig 9 (a–d).

Cox regression analysis demonstrated that risk of failure was approximately 8 times higher for overlays than onlays (p = 0.023; p < 0.05). Additionally, the incidence of fracture was approximately 4 times higher for restorations with one-sided PBE on the mesial or distal aspect compared to restorations with two-sided PBE on both approximal aspects (p = 0.047; p < 0.05). No statistically significant effect of endodontic treatment and periodontal status on treatment outcome was found (Table 5).

**Table 5:** The Cox regression analysis of the influence of the baseline variables on the failure outcomes

Variables		HR	95% CI		p-value
			Lower	Upper	
Gender	Female	3.717	0.446	31.008	0.225
	Male	Reference			
Age	+1 year	1.113	1.012	1.224	0.027
	-1 year	Reference			
Cuspal coverage	Overlay	8.554	1.342	52.522	0.023
	Onlay	Reference			
Endodontic treatment	Yes	1.702	0.328	8.840	0.527
	No	Reference			
Subgingival extension of restoration	Grade 3	1.971	0.450	8.629	0.368
	Grade 2	0	0	-	0.980
	Grade 1	Reference			
PBE sides	One-sided	4.905	1.021	23.574	0.047
	Two-sided	Reference			

HR: hazard ratio, CI: confidence interval.

Failures were repaired and excluded from following evaluation. However, they remained in the study recall for observation of the clinical outcomes of repair option. Five of 9 repaired PIRC restorations fractured again (case numbers of repeated fractures are given in Fig 1). Two (case numbers 47 and 48) of 9 fractures were observed in the same patient with prominent masseters but no sign of attrition or abfraction.

## Discussion

This study evaluated the clinical outcome of laboratory-processed PIRC restorations (SR Nexco) with PBE technique. Moreover, an attempt was made to analyse the reasons for failures. No significant difference was found for surface luster, surface staining, patient’s view, occlusal wear, color match and translucency scores, whereas acceptable (score 1–3) changes related to marginal quality, contact point and post-operative sensitivity were observed over time. Thus, the first null hypothesis, that there would be no differences in the FDI scores regarding the quality of the restorations compared to baseline, was rejected.

The survival (93.7%) and success (87.5%) rates obtained in the present study were in accordance with the survival rate of 91.1–94% and success rate of 84.8% in previous retrospective studies evaluating PIRC restorations with immediate dentin sealing (IDS) or PBE.^4,5^ A higher survival rate (100%) and success rate (96%) were obtained in the study by Dias et al,^8^ who evaluated resin composite overlays with PBE if required and IDS. The main reason for failure was fracture, but only one instance of secondary caries was observed and no debonding was reported in this previous prospective study.^8^ These findings corroborate with the results of the present study. Similarly, debonding was not a reason for failure of partial indirect restorations made of either ceramic or composite with PBE or IDS in previous studies.^2,4,29^ In contrast, debonding was shown to be the main reason for failure of indirect restorations without PBE or IDS in some previous studies.^13,35^ Considering these findings, it seems that IDS and PBE might have a positive influence on adhesion quality, as shown in previous in-vitro studies.^6,20,26^

The log-rank (Mantel-Cox) test did not show a statistically significant effect of any of the baseline variables on survival of the PIRC restorations. Thus, the second null hypothesis – that there would be no difference in survival of the PIRC restorations treated with one-sided or two-sided PBE, partial (onlay) or full cuspal coverage (overlay), endodontically treated or vital teeth, or a periodontal status of grade 1, 2 or 3 – was accepted. This result was consistent with the results of Van Den Breemer et al,^29^ who found no influence of endodontic treatment or number of cusps/surfaces involved on the survival of partial ceramic restorations with IDS. Similarly, Dias et al^8^ reported no statistically significant difference between the survival rates of two-sided or three-sided indirect resin composite restorations.

In the present study, the Cox regression analysis demonstrated that the risk of failure was higher for PIRC restorations with full cuspal coverage and one-sided PBE compared to PIRC restorations with partial cuspal coverage and two-sided PBE. The failures mainly observed within the 1-year observation period might be considered as technical failure rather than a consequence of fatique.^16^ The fractures observed in the short timeframe might be related to mechanical properties of the indirect resin composite, which might not have been able to resist occlusal forces in cavities with extensive substance loss (including all cusps), due to lack of reinforcement from the remaining tooth structure.^23^ Nevertheless, the transfer of occlusal forces to tooth structure exceeding the load-bearing capacity of the PIRC restorations seemed unlikely to lead catastrophic fractures. In contrast, Fennis et al^13^ reported cohesive failure as being one of the main reasons for failure for PIRC restorations without IDS or PBE; tooth integrity was maintained in the present study except in one case with reparable wall fracture. This contradictory finding might be attributed to the resin composite layer beneath the indirect resin composite restoration acting as a protective barrier against the stress of compressive forces. Previously, Van Den Breemer et al^30^ also suggested that the weakest link remained in IDS-cement-indirect restoration complex, based on the finding that the IDS layer was left adhered on the tooth surface after fracture. Contrary to the results of previous in-vitro studies showing no stastistically significant effect of PBE on the fracture strength of indirect restorations,^3,21^ the clinical outcome in the present study was more favorable when PBE was used at both mesial and distal cervical margins. However, no firm evidence could be provided for a beneficial effect PBE on fracture behavior of the restorations based on this finding, as solely PIRC restorations with different levels of subgingival extension of PBE (grades 1, 2 and 3) were evaluated in the present study.

Failure risk for endodontically treated teeth did not differ stastistically significantly from vital teeth in the present study. This finding was in accordance with the results of the studies using PBE or IDS with partial indirect restorations.^4,29^ Conversely, previous studies that did not using IDS showed more favorable outcomes for ceramic onlays placed in vital teeth compared to endodontically treated teeth.^28,31^

Contact points were scored as excellent immediately after placement of each restoration. However, three cases showed acceptable loss of contact at 6 months which disappeared at the 1-year recall. It was possible to annually evaluate two of these restorations up to 3 years; one remained stable, whereas the other relapsed after 30 months. Acceptable contact loss for partial indirect restorations was also reported by other authors to be within the range of 10.7–40%.^4,14,35^

The vast majority of the restorations evaluated after 3 years were scored as excellent for esthetic properties according to FDI criteria. Similar results relevant to color match were observed in previous studies,^7,15^ whereas minor roughness was shown to stastistically significantly increase in the study by Derchi et al.^7^ This contradictory finding might be related to different physical properties of the materials used. The UDMA-based resin composite microfilled with 10- to 50-nm silicon dioxide particles used in the present study might have been more resistant to degradation and wear over time, compared to the material with bis-GMA and TEG-DMA resin matrix and 1-µm particles in the study by Derchi et al.^7^ Notably, in an earlier study, an alpha score for surface texture after 3 years was given to 94.6% of the CAD/CAM generated resin composite inlays, which contained ultrafine-zirconia silica ceramic fillers in a bis-GMA and TEG-DMA resin matrix.^12^ Considering these findings, the type and size of the filler of the resin material seems to play an important role in durability of the surface texture of the PIRCs.

In a systematic review, minor deficiencies of marginal quality were reported to be in a range of 6.9–86.7%.^6^ Slight ditching and marginal discolorations might be accepted as a normal phenomenon due to degradation and wear of the luting composite over time. Notably, the findings of the study by Guess et al^17^ using the same adhesive were in accordance with the results of the present study regarding marginal discoloration and marginal adaptation within the same observation period. Considering these similar findings, the slight marginal deficiencies in the present study might be related to the technical operative procedure of the adhesive system. The highly viscous adhesive applied might not be distributed well and probably the areas with a thicker layer remained susceptible to degradation and resulted in small, localized areas of deterioration.

To the best of our knowledge, this study was the first prospective clinical trial evaluating PIRC restorations in conjunction with PBE. Based on the results of the study, PIRC restorations with PBE may represent a reliable option for cavities with extensive substance loss, considering the high survival rate and reparable failures. Given the results of the above-mentioned studies and the present study, it seems that PBE might have a beneficial effect on improved adhesion and reparable fractures. However, further randomised controlled clinical trials comparing PIRC restorations with or without PBE would be advisable to provide firm evidence. Additionally, esthetic properties and marginal quality of the PIRC restorations were satisfactory and remained stable after 3 years of observation. The risk of failure seemed to be less likely for onlay restorations covering two cusps compared overlay restorations. Considering the findings of previous studies and observations in the present study, contact loss related to tooth migration was a not entirely unexpected outcome. Based on our findings, follow-up might be recommended instead of immediate intervention to re-establish normal contact points.

The strictly followed protocol, well-defined size and extension of the indirect restorations were the strengths of the study. The single operator could be considered as one of the limitations of the study, since variability among operators was disregarded. However, the main limitation of this study was that not all of the restorations could be evaluated at 3 years. The enrollment phase was longer than the initially estimated period of one year, due to strict inclusion criteria. Although the sample size might have been higher, the number of restorations evaluated after 3 years was consistent with the initially defined sample size. Moreover, the observation period of 3 years with 5 recall sessions seemed to be adequate to evaluate the survival of restorations. Analysis of failure risk factors revealed important clinical implications for this treatment concept, involving the challenge presented by large cavities extending below gingiva. However, further studies with a larger sample and longer observation period are required to show degradation-related clinical outcomes over time.

## Conclusions

The survival and success rates of the PIRC restorations in conjunction with PBE was satisfactory, and clinically acceptable changes regarding the quality of surviving indirect restorations in terms of esthetic, functional and biological properties were observed over time. No difference was found in survival of the PIRC restorations treated with one-sided or two-sided PBE, partial (onlay) or full cuspal coverage (overlay), endodontically treated or vital teeth, or periodontal status (grades 1, 2, or 3). Risk of failure was less likely for PIRC restorations with partial cuspal coverge (onlays) compared to full cuspal coverage (overlays).

## Clinical relevance:

Restoration of teeth with extensive substance loss below the CEJ is challenging for clinicians. This study has provided evidence for the clinical applicability of indirect resin composite restorations with PBE to restore these large cavities, especially when partial cuspal coverage is indicated.
